# Supplementary material for: The mechanism of RNA duplex recognition and unwinding by DEAD-box helicase DDX3X
Source: Nat Commun. 2019 Jul 12;10:3085. doi: 10.1038/s41467-019-11083-2 (PMC6626043; doi:10.1038/s41467-019-11083-2)
Supplement: Supplementary file 1 — Supplementary Information [file 41467_2019_11083_MOESM1_ESM.pdf]

Supplementary Information

The mechanism of RNA duplex recognition and unwinding by DEAD-box helicase DDX3X

He Song and Xinhua Ji

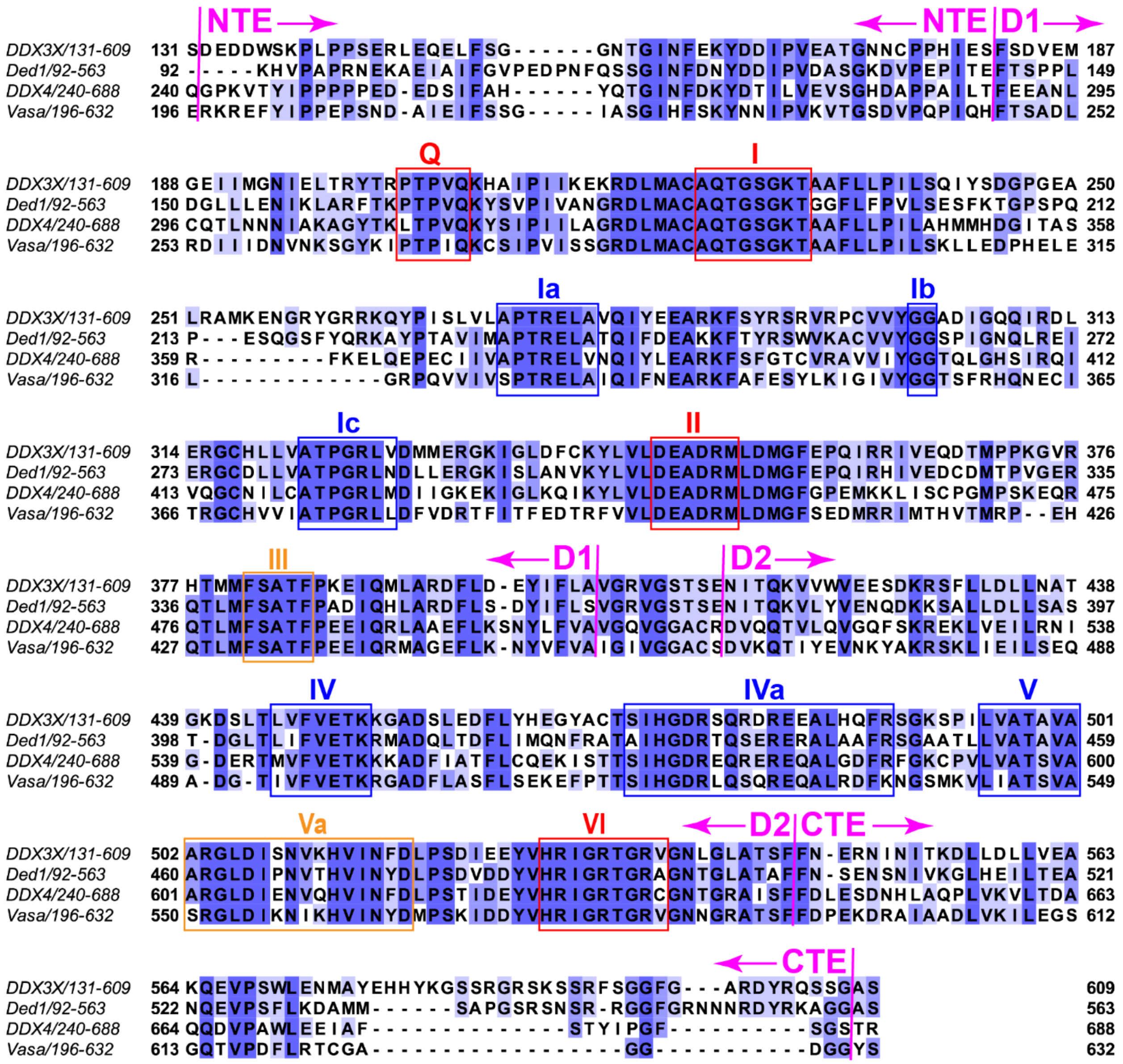

Supplementary Figure 1 | Sequence alignment of founding members of the DDX3X/Ded1 and DDX4/Vasa subfamilies of DEAD-box RNA helicases. Identical residues and similar residues are shaded in blue and light blue, respectively. Boundaries of the NTE (N-terminal extension), D1 (RecA-like domain 1), D2 (RecA-like domain 2), and CTE (C-terminal extension) are indicated. The 12 highly conserved sequence motifs are boxed, indicated, and color coded: red, ATP binding and hydrolysis; blue, RNA binding; and orange, coordination between ATP and RNA binding.

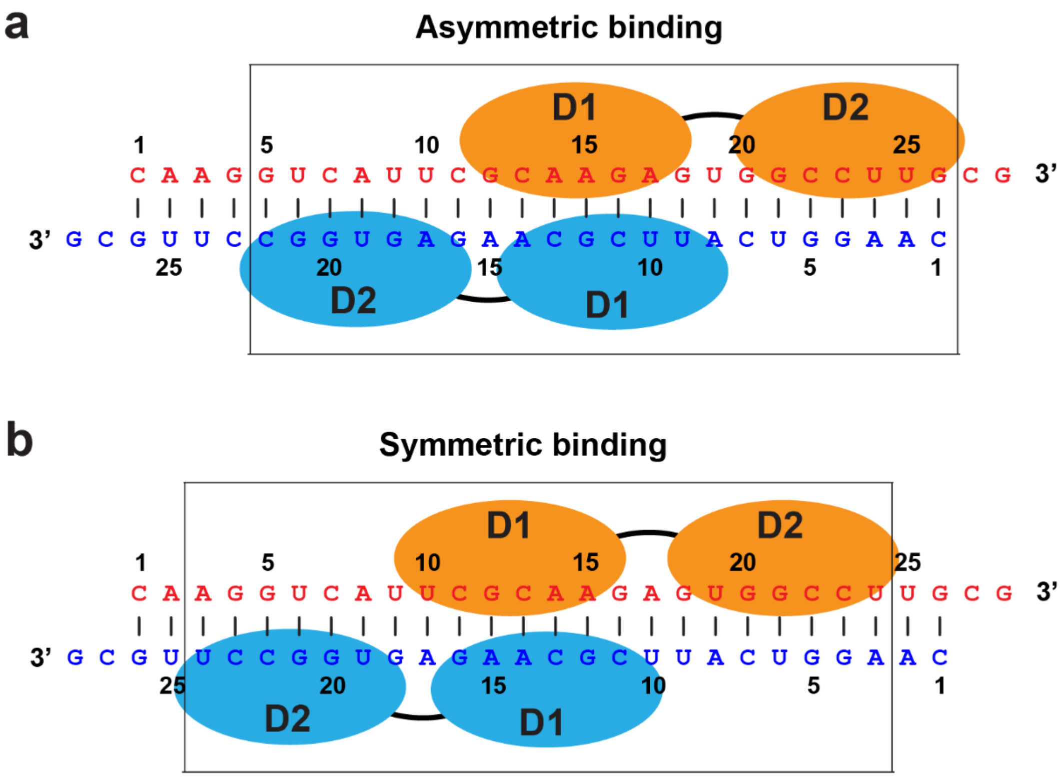

Supplementary Figure 2 | RNA duplex recognition by human DDX3X. a, Asymmetric binding of dsRNA by the D1D2 core of human DDX3X as observed in the D1D2:dsRNA:D1D2 structure. The D1D2 core contains amino acid residues 132-607 of human DDX3X (Fig. 1a) and the RNA contains 26 base pairs with a 2-nucleotide 3′ overhang at both terminal ends. The asymmetric binding results in different sequences for the two bound RNA strands. **b,** Symmetric binding of the dsRNA by the two D1D2 cores as imagined would results in the same sequence for the two bound RNA strands.

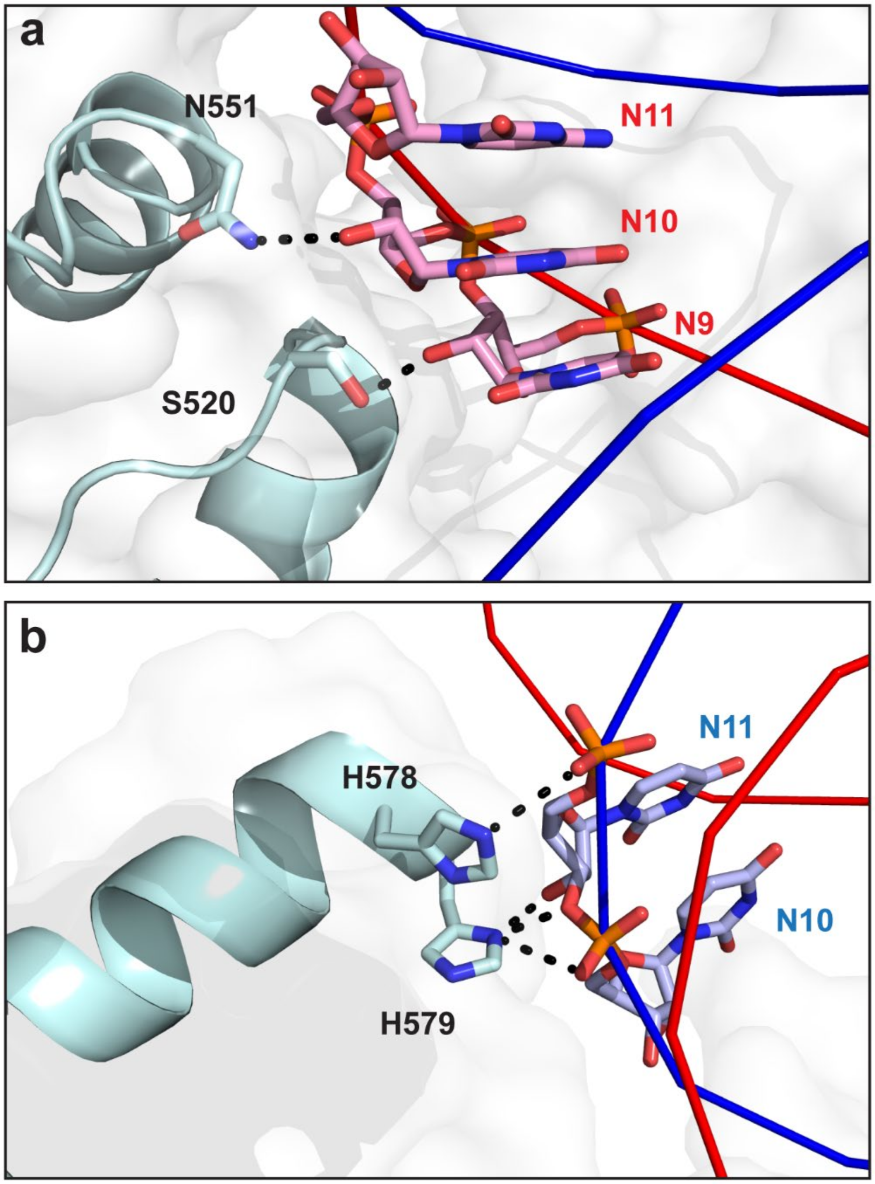

Supplementary Figure 3 | Detailed interactions between the CTE of human DDX3X and dsRNA. **a,** S520 and N551 of CTE recognize the 2′-hydroxyl groups of nucleotide residues 9 and 10 in the central region of RNA Strand 2 (backbone in red). **b,** H578 and H579 of CTE recognize the nucleotide residues 10 and 11 in the central region of RNA Strand 1 (backbone in blue).

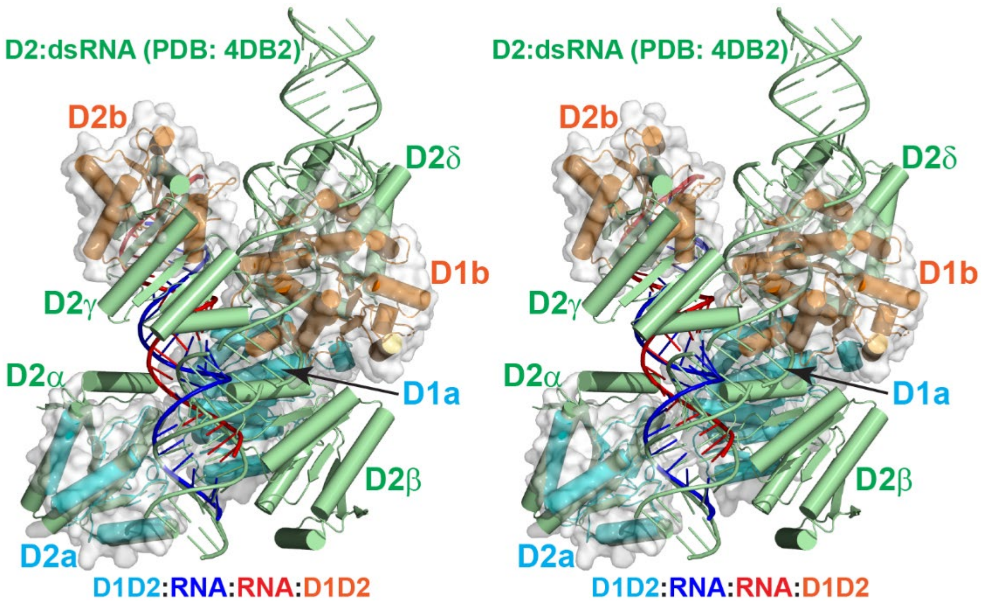

Supplementary Figure 4 | The DDX3X D1D2:dsRNA:D1D2 assembly vs the asymmetric unit content of the Mss116p D2:dsRNA structure in stereo. The D1D2:dsRNA:D1D2 assembly of DDX3X (this work), which is revealed in crystal, also observed in solution, and validated by Hill kinetics data, is illustrated as a cartoon model in cyan for D1D2^{MolA}, orange for D1D2^{MolB}, blue for RNA Strand 1, and red for RNA Strand 2. The two D1D2 cores are also outlined with transparent molecular surfaces. The asymmetric unit of the Mss116p D2:dsRNA structure, containing four D2s (D2α through δ) and a pseudo-continuous RNA duplex (the D2:D2:pseudo-dsRNA:D2:D2 ensemble, PDB: 4DB2) is shown as a cartoon model in palegreen. The two structures are superpositions on the bases of D2a in the D1D2:dsRNA:D1D2 assembly and D2α in the D2:D2:dsRNA:D2:D2 ensemble.

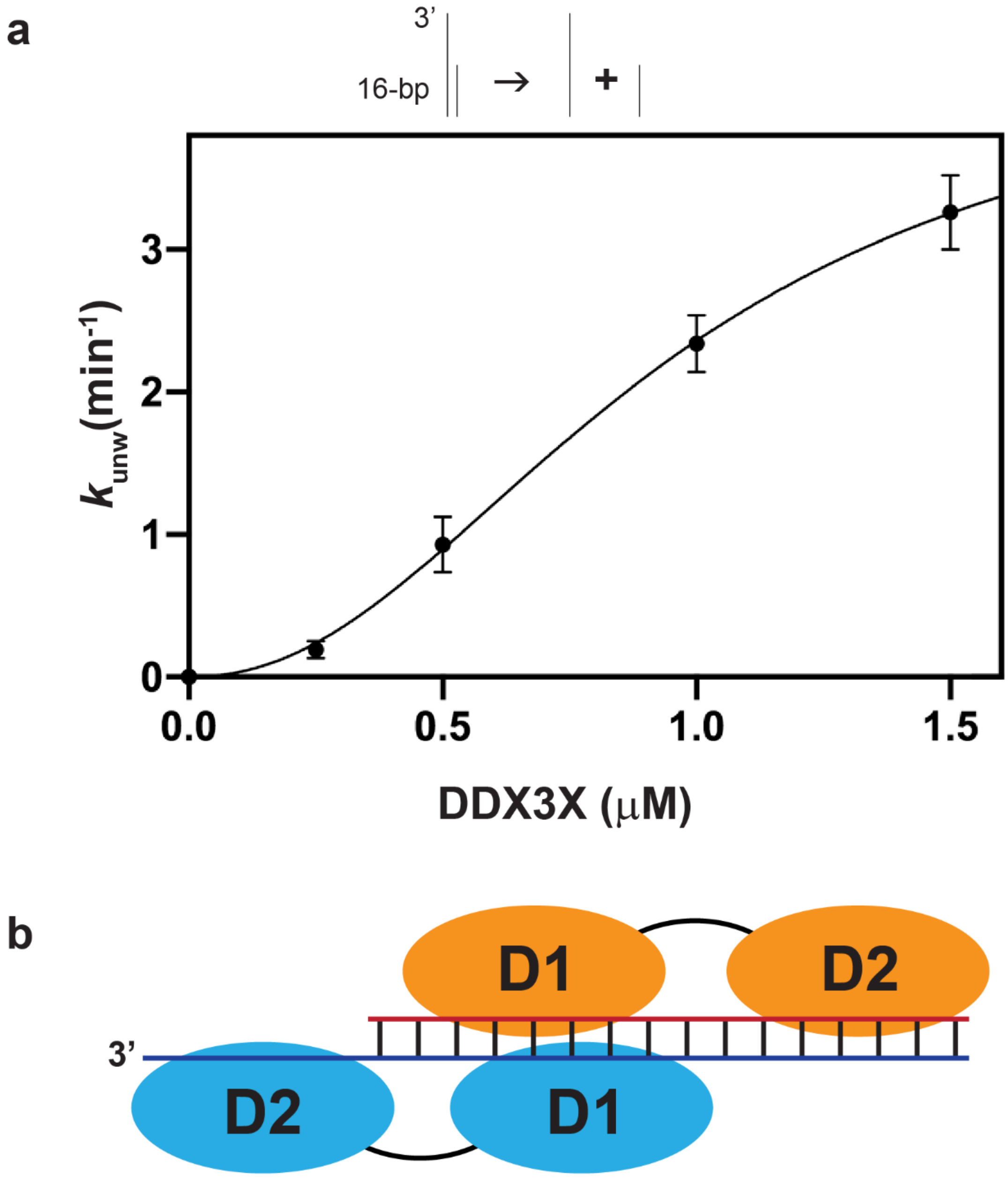

Supplementary Figure 5 | Hill kinetics for the unwinding activity of human DDX3X. a, Functional binding isotherm for unwinding the 16-bp dsRNA with a 25-nt 3′ overhang (Strand 1: 5′-GCGUCUUUACGGUGCUUAAAACAAAACAAAACAAAACAAAA-3′. Strand 2: 5′-AGCACCGUAAAGACGC-3′). Reactions contain 1 nM dsRNA with strand 2 labelled with biotin. Solid line is the best fits to the Hill equation (k_{unw}^{max} = 4.5 ± 0.7 min^{-1}, $K_{1/2}$ = 950 ± 150 nM, H = 2.1 ± 0.3). Error bars represent 1 SD. Source data are provided as a Source Data file. **b,** Schematic representation for the binding of the 16-bp dsRNA with 3′ overhang by two D1D2 cores of DDX3X.

Supplementary Table 1. Data-collection and scattering-derived parameters

Data-collection Parameters	
Beam line	12-ID-B (Advanced Photon Source)
Wavelength (Å)	0.8856
Detector	Pilatus 1M (SAXS), Pilatus 100K (WAXS)
q range ($Å^{-1}$)	0.006-2.8
Exposure time (s)	2
Concentration range (mg/ml)	1-4
Temperature	300
Structural Parameters	
$I(0)$ from Guinier fitting	0.309 ± 0.003
R_g from Guinier fitting (Å)	33.8 ± 0.3
$I(0)$ from GNOM	0.292 ± 0.004
R_g from GNOM (Å)	33.8 ± 0.3
D_{max} (Å)	105 ± 5
[a]MW^{pred} (kDa)	124
[b]MW^{saxs} (kDa)	120
Fit χ^2 value	1.72
Software Employed	
Primary Data Processing	Igor Pro
PDDF	GNOM
Ab initio Shape Analysis	DAMMIN
SAXS Profile Computation	CRYSOL
Superposition and averaging	SUPCOMB/DAMAVER
Molecular Visualization	PyMol

[a] The MW^{pred} was calculated from the primary sequence.

[b] The MW^{saxs} was calculated using the web portal "SAXS MoW".
